# Effectiveness of Dettol Antiseptic Liquid for Inactivation of Ebola Virus in Suspension

**DOI:** 10.1038/s41598-019-42386-5

**Published:** 2019-04-29

**Authors:** Todd A. Cutts, M. Khalid Ijaz, Raymond W. Nims, Joseph R. Rubino, Steven S. Theriault

**Affiliations:** 1 Applied Biosafety Research Program, Canadian Science Centre for Human and Animal Health, 1015 Arlington Street, Winnipeg, MB R3E 3P6 Canada; 2 J. C. Wilt Infectious Diseases Research Centre, Public Health Agency of Canada, 745 Logan Street, Winnipeg, MB R3E 3L5 Canada; 3 Research & Development, RB, One Philips Parkway, Montvale, New Jersey 07645 USA; 4 Medgar Evers College of the City University of New York (CUNY), 1650 Bedford Ave, Brooklyn, New York 11225 USA; 5 RMC Pharmaceutical Solutions, Inc., 1851 Lefthand Circle, Suite A, Longmont, Colorado 80501 USA; 6 Department of Microbiology, The University of Manitoba, Winnipeg, MB R3T 2N2 Canada

**Keywords:** Antivirals, Ebola virus

## Abstract

The efficacy of Dettol Antiseptic Liquid (DAL) for inactivating Ebola virus (Makona C07 variant) (EBOV/Mak) within an organic load in suspension was evaluated per ASTM E1052-11. Three DAL lots were evaluated at dilutions of 1:10, 1:20, and 1:40, with contact times of 0.5, 1, 5, and 10 min. Approximately 7 log_10_ tissue culture infectious dose_50_ (TCID_50_) of EBOV/Mak was exposed to DAL at ambient temperature. Each dilution tested reduced the infectious EBOV/Mak titer by ~5 log_10_ within one min. Detectable virus was still present after an 0.5-min exposure, but each DAL dilution caused >4 log_10_ reduction within this time. Detection of virus below the limit of detection of the TCID_50_ assay was assessed by inoculating flasks of Vero E6 cells with undiluted neutralized sample and evaluating the cultures for cytopathic effect after 14 days incubation. No infectious virus was detected with this non-quantitative method in samples subjected to DAL for 5 or 10 min, regardless of the dilution evaluated. The rapid and substantial inactivation of EBOV/Mak by DAL suggests that use of this hygiene product could help prevent the spread of Ebola virus disease during outbreaks.

## Introduction

The Ebola virus continues to re-emerge in lethal outbreaks, the most recent occurring in the Democratic Republic of the Congo, Africa in May 2018^1^. As of December 11, 2018, the World Health Organization reported that the Ebola outbreak in the North Kivu and Ituri provinces of the DRC has included a total of 505 cases, with 457 confirmed and 48 probable, and has resulted in 296 deaths^2^. Overall, to date there have been 34 Ebola virus disease outbreaks, 18 of which have involved Zaire ebolavirus since the initial emergence of this strain in 1976. This most recent outbreak, and the outbreaks occurring between 2014–2016, have refocused efforts of public health agencies such as the World Health Organization^2^ on identifying approaches to reduce the spread of the disease from community to community and from nation to nation. Ebola virus disease is included in the World Health Organization’s List of Blueprint Priority Diseases^3^, a list of diseases for which: “… given their potential to cause a public health emergency and the absence of efficacious drugs and/or vaccines, there is an urgent need for accelerated research and development…”^3^.

It is known that the Ebola virus may be transmitted by contact with infected corpses, infected environmental surfaces (fomites), and secretions and excretions from infected individuals^4^. It has also been shown that fomites in the vicinity of infected patients may be contaminated with Ebola virus RNA^5^. Environmental persistence of infectious EBOV Makona (EBOV/Mak) suspended in an organic soil load have been reported at eight days from experimentally contaminated surfaces^6^. An important intervention approach might therefore involve the use of an effective virucidal agent for disinfecting surfaces and spills potentially contaminated with Ebola virus, thereby mitigating the risk of transmission to healthy individuals, including health-care providers.

There are relatively little suspension inactivation data for the Ebola virus itself. The efficacies of microbicides (disinfectants and antiseptics) for inactivation of Ebola virus typically been determined through evaluation of the inactivation, by such products, of appropriate surrogate viruses such as bacteriophages, enveloped viruses (animal coronaviruses, influenza viruses), or non-enveloped viruses such as caliciviruses or picornaviruses. The Ebola virus is a member of the *Filoviridae* family, and being an enveloped virus should be relatively susceptible to a variety of microbicidal inactivation approaches. In view of the lethality of the virus, however, the United States Centers for Disease Control and Prevention (CDC) has provided the following guidance^7^: “… selection of a disinfection product with a higher potency than what is normally required for an enveloped virus is being recommended at this time. EPA-registered hospital disinfectants with label claims against non-enveloped viruses (noroviruses, rotavirus, adenovirus, poliovirus) are broadly antiviral and capable of inactivating both enveloped and non-enveloped viruses.” Per the United States Environmental Protection Agency (EPA), in order to claim efficacy of a product for an emerging enveloped virus, the product needs to be approved by EPA for inactivating at least one large or one small non-enveloped virus^8^.

Efficacy testing of microbicides through the study of inactivation of surrogate non-enveloped viruses theoretically should ensure their efficacy for inactivation of the Ebola virus. However testing conducted specifically with the high-risk pathogens including viruses is also needed, to provide assurance to critical facilities and personnel. In the present effort, efficacy studies were performed at the Canadian Science Centre for Human and Animal Health Biosafety Level 4 (BSL-4) facility.

In the present study, we evaluated the efficacy of a commercially available hygiene product, Dettol Antiseptic Liquid (DAL), for inactivating EBOV/Mak is suspension. Dettol is used in Europe, Africa, and Asia in homes and healthcare settings for various first aid antiseptic purposes, including wound cleansing^9^. It is also used for personal hygiene purposes, and as a microbicide for decontaminating environmental surfaces, objects, and equipment. The microbicidal active ingredient in DAL is 4-chloro-3,5-dimethylphenol (chloroxylenol), also known by its non-systematic name para-chloro-meta-xylenol or PCMX^10^. The concentration of the active in DAL is 4.8% (weight to volume) PCMX. Three concentrations each of DAL lots 15083E, 16004E, and 16005E were evaluated for inactivation of EBOV/Mak suspended in an organic soil load using the method specified in the ASTM 1052-11 international standard^11^. This test method was developed by the American Society for Testing and Materials International (ASTM) to standardize the evaluation of virucidal activity of microbicidal products in suspension. Organic soil loads are added to the study design in order to better model viral inactivation by microbicides in relevant matrices such as human sputum or blood. Use of hard water as diluent was included in the study design to simulate water hardness in the field. Four contact times (0.5, 1, 5, and 10 min) were evaluated in triplicate for each of three independent lots of DAL. In addition to the methodology described in the ASTM standard, we also evaluated any residual infectious virus following exposure to DAL through inoculation of 500 µL of undiluted neutralized test sample into T-75 flasks of Vero E6 indicator cells. This was done to evaluate the possibility of virus being present at levels lower than the limit of detection of the tissue culture infectious dose_50_ (TCID_50_) assay performed in Vero E6 cells per the ASTM standard.

## Results

### Neutralization Effectiveness Evaluation

During the evaluation of possible neutralizing agents, it was determined that 100% fetal calf serum (FCS) and 100% virus culture medium (VCM) failed to adequately terminate the viral inactivating effects of DAL. On the other hand, 1× Letheen broth in VCM (10× Letheen broth diluted 1:10 in VCM), added to the DAL dilutions prior to introduction of the EBOV/Mak in tripartite soil load^12,13^, prevented inactivation of the virus. As shown in Supplemental Figs S1 and S2, no statistically significant (*P* < 0.05) differences in the viral titers obtained for the virus positive controls, the virus + DAL dilution + neutralizer, and virus + neutralizer conditions were observed when 1× Letheen broth was evaluated. The disinfectant neutralizing agent that was used in each of the inactivation efficacy studies described below was 1× Letheen broth.

### Virucidal Efficacy Results

**Figure 1:**
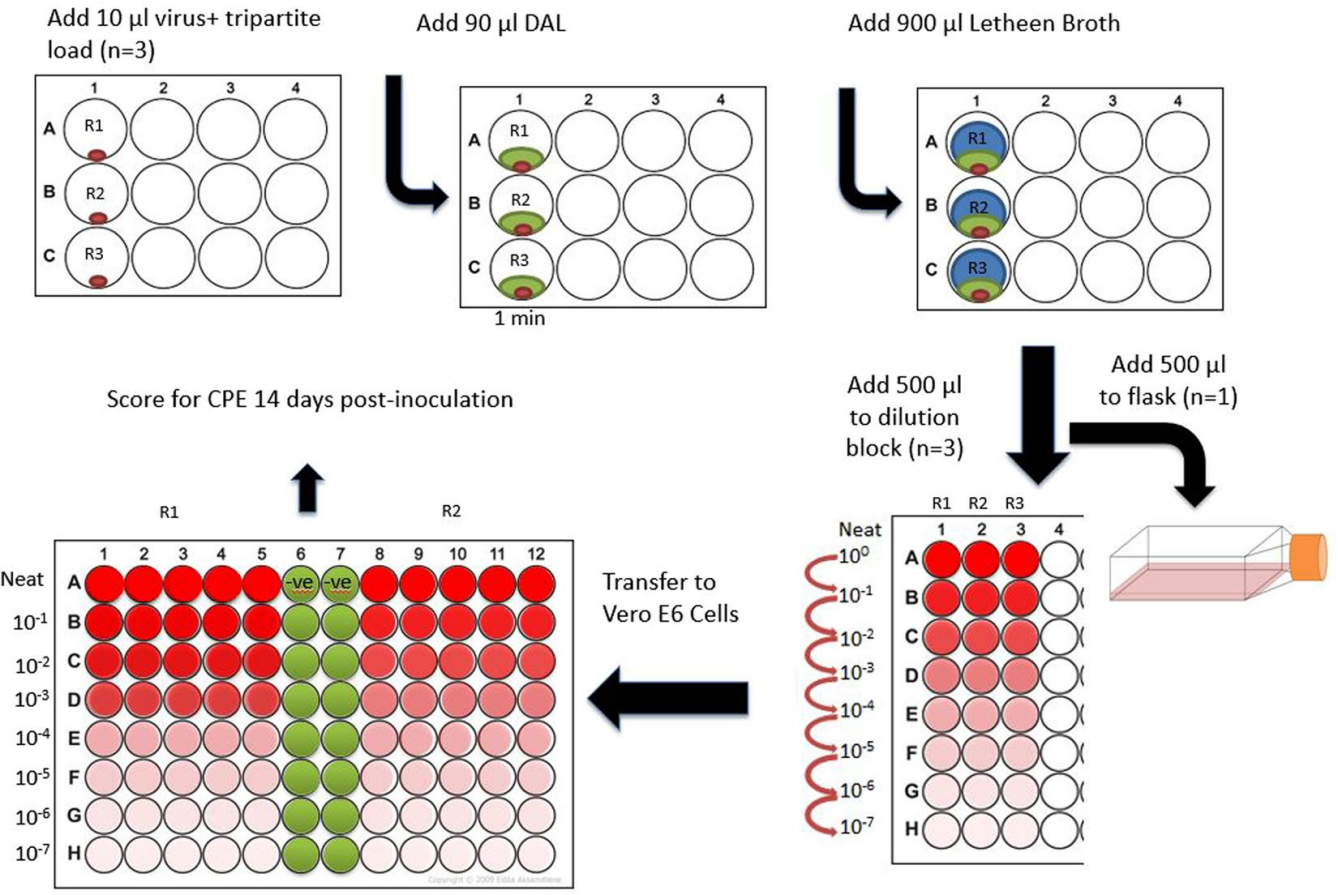
Schematic representation of the inactivation efficacy testing methodology employed. The entire procedure was performed once for each DAL lot.

Three different lots of DAL were evaluated at three dilutions of DAL each (1:10, 1:20, and 1:40 in hard water, corresponding to 0.48%, 0.24%, and 0.12% of PCMX active, respectively) in triplicate. Contact times of 0.5, 1, 5, and 10 min at ambient temperature were evaluated in a BSL-4 facility. An initial EBOV/Mak titer of 1.7 × 10^8^ log_10_ TCID_50_/mL in tripartite soil load was exposed to the various DAL dilutions and contact times using the methodology depicted in Fig. 1. The post-exposure/neutralization titers for the positive virus controls (virus alone) and the DAL test conditions were calculated. The log_10_ reduction values for each time point were calculated by subtracting the titers obtained for the DAL test conditions from the titers of the corresponding positive virus controls.

**Figure 2:**
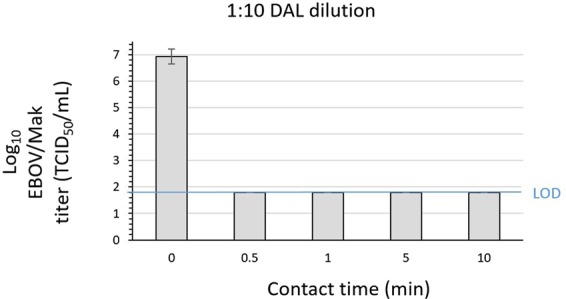
Time kinetics for EBOV/Mak inactivation efficacy results for the 1:10 dilution of DAL lots 15083E, 16004E, and 16005E at ambient temperature. The values represent the mean ± standard deviation (n = three replicates, one for each DAL lot) of the log_10_ titer of the positive control (0 minutes contact time) and the post-neutralization samples (0.5, 1, 5, and 10 minutes contact time). Individual viral titers were calculated based on three replicate wells per dilution. The limit of detection (LOD) of the assay was 1.8 log_10_ TCID_50_/mL (shown in the plot as a blue line extending from y = 1.8 log_10_ TCID_50_/mL). This was due to the cytotoxicity of the DAL dilution to the Vero E6 cells.

For the 1:10 dilution of the three DAL lots at 0.5, 1, 5, and 10 min contact time, the mean ± standard deviation values for the log_10_ EBOV/Mak titers measured for the positive virus control condition (virus alone) and the post-exposure titers are displayed in Fig. 2. Complete inactivation (4.8 to 5.4 log_10_) of EBOV/Mak to the limit of detection of the assay (1.8 log_10_ TCID_50_/mL virus) was observed for all replicates and contact times.

**Figure 3:**
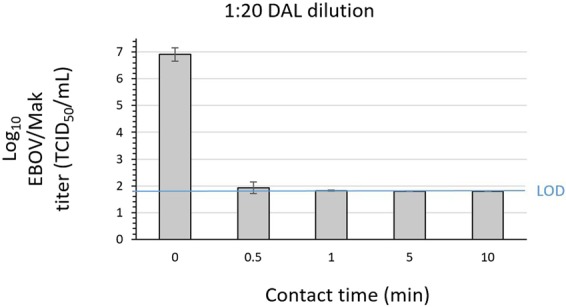
Time kinetics for EBOV/Mak inactivation efficacy results for the 1:20 dilution of DAL lots 15083E, 16004E, and 16005E at ambient temperature. The values represent the mean ± standard deviation (n = three replicates, one for each DAL lot) of the log_10_ titer of the positive control (0 minutes contact time) and the post-neutralization samples (0.5, 1, 5, and 10 minutes contact time). Individual viral titers were calculated based on three replicate wells per dilution. The limit of detection (LOD) of the assay was 1.8 log_10_ TCID_50_/mL (shown in the plot as a blue line extending from y = 1.8 log_10_ TCID_50_/mL). This was due to the cytotoxicity of the DAL dilution to the Vero E6 cells.

For the 1:20 dilution of the three DAL lots at 0.5, 1, 5, and 10 min contact time, the mean ± standard deviation values for the log_10_ EBOV/Mak titers measured for the positive virus control condition (virus alone) and the post-exposure titers are displayed in Fig. 3. Virus was detected at the 0.5 min contact time for DAL lot 16004E and at the 1 min time for DAL lot 15083E. Complete inactivation (4.8 to 5.3 log_10_) of EBOV/Mak to the limit of detection of the assay (1.8 log_10_ TCID_50_/mL virus) was observed for all replicates following contact times of 5 min or greater.

**Figure 4:**
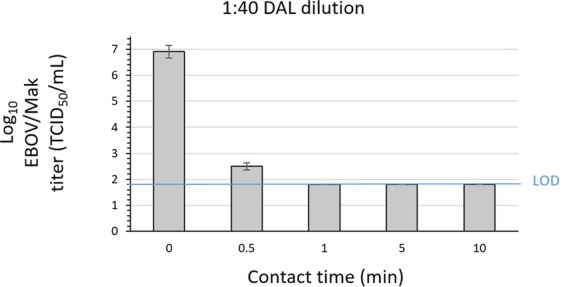
Time kinetics for EBOV/Mak inactivation efficacy results for the 1:40 dilution of DAL lots 15083E, 16004E, and 16005E at ambient temperature. The values represent the mean ± standard deviation (n = three replicates, one for each DAL lot) of the log_10_ titer of the positive control (0 minutes contact time) and the post-neutralization samples (0.5, 1, 5, and 10 minutes contact time). Individual viral titers were calculated based on three replicate wells per dilution. The limit of detection (LOD) of the assay was 1.8 log_10_ TCID_50_/mL (shown in the plot as a blue line extending from y = 1.8 log_10_ TCID_50_/mL). This was due to the cytotoxicity of the DAL dilution to the Vero E6 cells.

For the 1:40 dilution of the three DAL lots at 0.5, 1, 5, and 10 min contact time, the mean ± standard deviation values for the log_10_ EBOV/Mak titers measured for the positive virus control condition (virus alone) and the post-exposure titers are displayed in Fig. 4. Surviving virus was detected at the 0.5-min contact time for each of the three DAL lots. Complete inactivation (4.8 to 5.3 log_10_) of EBOV/Mak to the limit of detection of the assay (1.8 log_10_ TCID_50_/mL virus) was observed for all replicates following contact times of 1 min or greater.

The possible presence of infectious EBOV/Mak below the limit of detection of the TCID_50_ assay (1.8 log_10_ TCID_50_/mL) was evaluated by inoculating T-75 flasks of Vero E6 cells with 500 µL of undiluted neutralized test sample after each contact time and then evaluating the cultures microscopically for viral cytopathic effects (CPE) after 14 days of incubation. The results of this flask safety testing for the 1:10, 1:20, and 1:40 DAL dilutions (n = 1 flask per condition and DAL lot) are displayed in Table 1. Infectious EBOV/Mak was detected for the 1:10 dilution of DAL lot 16005E at the 0.5-min contact time. No infectious virus was detected for the 1:10 dilution of either DAL lot following contact times of 1 min or greater. The results of the flask safety testing for the 1:20 DAL dilution (n = 1 flask per condition and DAL lot) are also displayed in Table 1. Infectious EBOV/Mak was detected for DAL lots 15083E and 16005E at the 0.5-min contact time and for DAL lot 16004E at the 1-min contact time. No infectious virus was detected for the 1:20 dilution of the three DAL lots following contact times of 5 min or greater.

**Table 1:** Flask safety test results for Dettol Antiseptic Liquid (DAL) lot 1 (16004E), lot 2 (15083E), and lot 3 (16005E).

Test Condition (contact time)	1:10 DAL			1:20 DAL			1:40 DAL		
	Lot 1	Lot 2	Lot 3	Lot 1	Lot 2	Lot 3	Lot 1	Lot 2	Lot 3
Negative control	0	0	0	0	0	0	0	0	0
Neutralizer + DAL	0	0	0	0	0	0	0	0	0
DAL (0.5 min)	0	0	**+**	**+**	0	**+**	**+**	**+**	**+**
DAL (1 min)	0	0	0	0	**+**	0	0	0	0
DAL (5 min)	0	0	0	0	0	0	0	0	0
DAL (10 min)	0	0	0	0	0	0	0	0	0

+, Viral CPE observed; 0, viral CPE not observed, or no cytotoxicity observed for N + DAL; N + DAL, neutralizer + DAL (cytotoxicity) control. One replicate flask per DAL lot and test condition was evaluated.

The results of the flask safety testing for the 1:40 DAL dilution (n = 1 flask per condition and DAL lot) (Table 1) demonstrate that infectious virus was detected for each DAL lot at the 0.5-min contact time only. No infectious EBOV/Mak was detected for the 1:40 dilution of the three DAL lots following contact times of 1 min or greater.

## Discussion

Due to the need for a BSL-4 containment facility for conduct of inactivation studies on the Ebola virus, there is little information on the efficacy of microbicidal products for suspension inactivation of this filovirus. Efficacy data for microbicides against small or large non-enveloped viruses have been used in support of efficacy claims against emerging enveloped viruses such as Ebola virus^4,8,14^.

In view of the paucity of available information on suspension inactivation of Ebola virus and other hemorrhagic fever viruses, the CDC has recommended^15^ the use of a 1:10 dilution of household bleach (0.5% [5000 ppm] final chlorine concentration) for disinfection of infected excreta and corpses. Reproducible inactivation of viruses by chlorine is dependent upon free chlorine concentration, which is dependent both upon the chlorine demand of the inactivation solution as well as the free chlorine concentration of the bleach solution being used. The efficacy of free chlorine for inactivation of EBOV/Mak in sterilized municipal wastewater was investigated by Bibby *et al*.^16^. The inactivation resulting from 1 ppm chlorine at 20 °C was incomplete but substantial (3.5 log_10_ by 0.3 min contact time, but little additional activation was observed over contact times up to 60 min). Higher chlorine concentrations (5 and 10 ppm chlorine, corresponding to initial free chlorine concentrations of 0.5 and 1.1 ppm, respectively) resulted in complete inactivation of the EBOV/Mak (4.2 log_10_ reduction for 0.3 min contact time).

Bleach, while an effective microbicide, is not practical for use on intact or wounded human skin. In addition, as mentioned above, the free chlorine levels of commercial bleach products may vary and effect the final free chlorine of the final inactivation solutions. On the other hand, the halophenolic compound PCMX, which is the active microbicidal ingredient in DAL, is suitable for use for preoperative skin antisepsis^17^ and for disinfecting surgical instruments^10,18^. It is an active ingredient used in a number of household microbicidal products, including DAL. As with other phenolics, the mechanism of viral inactivation for PCMX is likely related to disruption of lipid envelopes^19^. The virucidal effectiveness of PCMX formulations is therefore expected to be greatest for enveloped viruses.

It has been proposed that the detergent Triton-X 100 be used at 0.1% to decontaminate clinical specimens (e.g., blood serum) in order to reduce (albeit not completely) the titer of Ebola virus in such specimens^20^. As is implicit in this guidance, the inactivation afforded by 0.1% Triton X-100 is not to be considered complete and, in fact, van Kampen *et al*.^21^ have shown that the percentage of serum in a test sample determines the extent of inactivation. In a sample matrix consisting of 98% fetal bovine serum, there was no statistically significant log_10_ reduction of Ebola virus resulted from a 1-hour exposure to 0.1% Triton X-100. A similar result was obtained when 0.1% sodium dodecyl sulfate was used as the inactivating agent. In samples containing lower percentages (9.8%, 0.98%) of fetal bovine serum, the inactivation caused by these agents was >3 log_10_, but still not complete. The inability of Triton X-100 at up to 1% to completely inactivate Ebola virus in blood serum has also been reported by Burton *et al*.^22^ and Colavita *et al*.^23^.

Efficacy for viral inactivation is typically expressed in terms of an expected log_10_ reduction value. For instance, the US Environmental Protection Agency (EPA) stated in its 2012 disinfectant product guidance^24^ that “The product should demonstrate complete inactivation of the virus at all dilutions. If cytotoxicity is present, the virus control titer should be increased to demonstrate a ≥3 log_10_ reduction in viral titer beyond the cytotoxic level.” For disinfectants that are non-cytotoxic to the cellular infectivity assays used for demonstrating efficacy, a 4-log_10_ reduction in viral titer is considered to be effective. These EPA requirements for demonstrating acceptable efficacy were recently modified in the 2018 revision^25^. In the revised guidance, a valid test requires (1) that at least 4.8 log_10_ of infectivity per carrier be recovered from the dried virus control film; (2) that a ≥3 log_10_ reduction in titer must be demonstrated in the presence or absence of cytotoxicity; (3) if cytotoxicity is present, at least a 3 log_10_ reduction in titer must be demonstrated beyond the cytotoxic level; and (4) that the cell controls be negative for infectivity. In the revised guidance, therefore, an efficacious product does not need to demonstrate complete inactivation at all dilutions.

The significance of Ebola virucidal efficacy determined in terms of a log_10_ reduction value must, however, be considered against the backdrop of the mortality associated with the virus, the estimated infectious dose, and the expected viral load within a contaminated bodily fluid. The mortality associated with Ebola virus disease is high. For example, the overall case fatality rate for outbreaks occurring between 1976 and 2017 has been calculated as 67%^26^. The infectious dose of Ebola virus is believed to be relatively low in humans (between 1 and 10 infectious units)^16,27^. The median plasma viral load in confirmed Ebola virus disease patients during the 2014–2015 Sierra Leone outbreak was found to be 6.7 log_10_ genomic copies per mL (range: 5.5 to 7.8 genomic copies/mL)^28^. The average peak blood serum titer in cases from the 2000–2001 Uganda outbreak with a fatal outcome was 3.4 × 10^9^ genomic copies per mL, corresponding to 3.4 × 10^5^ to 3.4 × 10^6^ infectious units per mL^29^. Human-to human transmission of Ebola virus disease has been attributed to direct contact with infected blood (including reuse of contaminated needles) or other secretions or bodily fluids of infected persons, and to contact with infected corpses^30,31^. For example, infected patients have been reported to produce large volumes (5 L or more) of watery diarrhea over a period of 7 days or longer^32^. The point of this discussion is to stress that the Ebola virucidal efficacy needs to be as high as possible, and certainly should be greater than the 3 or 4 log_10_ reduction routinely expected of microbicidal products.

Efficacy and lot-to-lot consistency of commercially available DAL for inactivating Ebola virus in suspension in the presence of a tripartite organic load has been demonstrated in the present studies. Due to the lethality of the Ebola virus and the low infectious dose for humans noted above, the studies were performed to determine log_10_ reduction in titer, as typically is done in demonstrating virucidal efficacy for microbicidal products. In addition, however, studies were conducted in a manner intended to assure that no infectious virus remained following application of the microbicidal product. The latter studies, referred to here as flask safety testing, was based on the assumption that any residual infectious viral particles following exposure to DAL should be able to infect the susceptible Vero E6 cells in the inoculated T-75 flasks and to amplify over a 14-day observation period, causing detectable viral CPE in the flasks. The greatest assurance of complete inactivation of EBOV/Mak was demonstrated for contact times of 5 min or greater (DAL dilutions of 1:20 and 1:40) or 1 min or greater in the case of the 1:10 DAL dilution. Under these conditions, over 5 log_10_ reduction in titer of infectious Ebola virus in tripartite soil load was demonstrated in the quantitative TCID_50_ assay and no detectable virus was detected in the non-quantitative flask safety tests.

These results suggest that DAL used at a 1:10 dilution in water should afford complete and rapid (within 1 min contact time) inactivation of EBOV/Mak in suspensions at ambient temperature. The data from the TCID_50_ assay and the flask safety testing performed for this DAL dilution made in hard water indicate that over 7 log_10_ inactivation of EBOV/Mak was achieved under these conditions.

## Materials and Methods

### Cell Line, Virus, and Medium

African green monkey Vero E6 cells (ATCC CRL-1586) were maintained at 37 °C/5% CO_2_ in Dulbecco’s modified Eagle medium (DMEM, HyClone) supplemented with 10% FCS (Gibco) and 10 units/mL penicillin/streptomycin (Gibco). Ebola virus (Makona C07 variant) (Ebola virus/H. sapiens-tc/GIN/2014/Makona-C05) was obtained from a clinical isolate. All manipulations involving EBOV/Mak were carried out in a BSL-4 laboratory at the Canadian Science Centre for Human and Animal Health, Winnipeg, Manitoba, Canada.

### Stock Virus Preparation

A characterized stock of EBOV/Mak was prepared by infecting ten T-75 flasks of African green monkey Vero E6 cells (ATCC CRL-1586) at ~80% confluency at a multiplicity of infection (MOI) of 0.01. At approximately 9 days post-infection, marked cytopathic effects were evident in the Vero E6 cells, at which time the flasks were frozen at −70 °C. The flasks were thawed the following day and the conditioned medium from the flasks was clarified by low-speed centrifugation (4500 × g) for 10 min. The supernatants were pooled and overlaid onto 20% w/v sucrose cushions prepared in Tris- NaCl-EDTA buffer. After centrifugation at ~130000 × g for 2 h, the resulting viral pellets were resuspended in VCM (DMEM containing 2% FCS and 10 units/mL penicillin/streptomycin) overnight at 4 °C. The resuspended virus was pooled and aliquoted into usable amounts and frozen at −70 °C until needed. Stock virus titers were determined to be >9 log_10_/mL by TCID_50_ assay, with titer calculation following the Reed-Muench method^33^.

### Dettol Antiseptic Liquid Product Dilution

Dilutions of DAL lot# 15083E, 16004E, and 16005E were prepared in hard water^12^ (prepared as 1 L deionized water supplemented with 0.4 g calcium carbonate) on the day of assay performance. The resulting solutions were inverted to mix and used within 2.5 to 4 h of preparation.

### Assessment of Product Neutralization

A neutralization assay was performed to evaluate the ability of candidate neutralizing reagents to neutralize the virucidal effects of DAL post-exposure to the neutralizers. The reagents evaluated included 100% FCS, VCM, 1× Letheen broth in VCM (10× Letheen broth, BD Difco, diluted 1:10 in VCM). Ebola virus (Makona C07 variant) was diluted to approximately 10^4^ to 10^6^ TCID_50_ per mL with 10 µL virus evaluated per control in replicates of three. The candidate reagents were evaluated for neutralization efficacy (by scoring wells for CPE) and cytotoxicity to Vero E6 cells (by microscopic evaluation of monolayers in wells for morphology and confluency of Vero E6 cells) using the following conditions:

#### Negative Control

Cells were cultured in VCM and used as a baseline of comparison for evaluation of cytotoxicity or CPE.

#### Neutralizer Control

The candidate neutralizers to be evaluated were diluted in VCM using a 10-fold serial scheme from 10^0^ (undiluted) to 10^−3^. Neutralizer dilutions were added (50 µL) to Vero E6 cells (n = 5 replicates per dilution). Cells were scored for cytotoxicity 14 days post-inoculation.

#### Positive Control

The virus control was prepared by adding 10 µL of EBOV/Mak in a tripartite soil load^12,13^ (10^2^ to 10^4^ TCID_50_ virus; 0.25% bovine serum albumin [BSA, Sigma], 0.35% tryptone [Becton Dickinson], 0.08% bovine mucin [Sigma]) to 990 µL of VCM. Final concentrations in the control were: virus (10^2^ to 10^4^ TCID_50_/mL), BSA (0.0025%), tryptone (0.0035%), and mucin (0.0008%). The positive control were diluted in VCM using a ten-fold dilution scheme from 10^0^ (undiluted) to 10^−3^, and 50 µL of the resulting solutions were added to Vero E6 cells. Cells were scored for CPE 14 days post-inoculation.

#### Neutralizer + Virus Controls

To account for the effect of the neutralizer acting on the virus, neutralizer + virus controls were prepared. These were prepared exactly the same way as the positive virus control, except that the 10 µL of virus in tripartite soil load was added to 990 µL of candidate neutralizer instead of VCM. The neutralizer + virus controls were diluted in VCM using a ten-fold dilution scheme from 10^0^ (undiluted) to 10^−3^, and 50 µL of the resulting solutions were added to Vero E6 cells. Cells were scored for cytotoxicity and CPE 14 days post-inoculation.

#### Neutralizer + Disinfectant + Virus Controls

To demonstrate the efficacy of the candidates for neutralizing viral inactivation by DAL, 50 µL of diluted DAL were added to 940 µL of the candidate neutralizers. Shortly thereafter, 10 µL of virus in tripartite soil load (concentrations of components given above) were added and the resulting mixtures (neutralizer + disinfectant + virus or controls), were diluted in VCM using a ten-fold dilution scheme from 10^0^ (undiluted) to 10^−3^, and 50 µL of the resulting solutions were added to Vero E6 cells. Cells were scored for cytotoxicity and CPE 14 days post-inoculation.

### DAL Efficacy Testing

Inactivation efficacy testing for DAL was performed in suspension studies (Fig. 1) conducted at ambient temperature per ASTM E1052-11^11^. Stocks of EBOV/Mak in tripartite soil load were prepared on the day of assay as follows. A single tube of stock virus was removed from frozen storage and mixed with a tripartite soil load^12,13^ (~1.7 × 10^7^ TCID_50_ virus, 0.25% bovine serum albumin, 0.35% tryptone, 0.08% mucin). The virus in tripartite soil load (10 µL) was added to 90 µL of diluted DAL or to 90 µL VCM (positive virus control). The resulting solutions were mixed and held at room temperature for 30 seconds, 1 min, 5 min and 10 min contact times. At the end of each exposure time point, the DAL was neutralized by adding 900 µL of prepared 1× Letheen broth to the test solutions and pipetted repeatedly to mix. A 500-µL portion of each neutralized test solution was diluted in VCM using a ten-fold dilution scheme, and 50 µL of the resulting dilutions were added to 96-well plates containing Vero E6 cells monolayers (n = 5 replicates per dilution). After a 45-min adsorption period, 150 µL of VCM were added to each well. The Vero E6 cell monolayers in the wells were scored 14 days post-infection for CPE and virus titers (TCID_50_) were calculated according to the Reed-Muench method^33^.

Since the undiluted post-neutralization DAL displayed cytotoxic effects in the Vero E6 cells and therefore could not be evaluated for viral inactivation, an alternative method (flask safety test) was employed to evaluate the undiluted test solutions for the presence of any remaining infectious EBOV/Mak. In this test, 500 µL aliquots of undiluted neutralized inactivation test solution were added to single T-75 flasks (n = 1 replicate per DAL lot and neutralization time point) of Vero E6 cells monolayers at ~80% confluency, along with 20 mL of VCM. The flasks were incubated at 37 °C/5% CO_2_ for 14 days and then scored for presence or absence of CPE.

In addition to the efficacy test conditions described above, a single cytotoxicity control was performed in parallel with each assay. This was done to control for observable CPE or cytotoxicity caused by the neutralizer and DAL dilution being tested. This involved combining 900 µL of 1× Letheen broth and 100 µL of diluted DAL. A 500-µL portion of the resulting solution was diluted in VCM using a ten-fold dilution scheme, and 50 µL of the resulting dilutions were added to 96-well plates containing Vero E6 cells (n = 5 replicates per dilution). The Vero E6 cell wells were scored for cytotoxicity 14 days post-inoculation.

### Analysis of Inactivation Efficacy

To score the test plates post-incubation, TCID_50_ titers for positive virus controls and neutralized DAL test conditions were determined by the method of Reed and Muench^33^. The log_10_ reduction values achieved by the various DAL lots and exposure time points were calculated by subtracting the post-virucidal efficacy test of DAL, log_10_ TCID_50_ values from the log_10_ titers obtained for the corresponding positive virus controls. Statistical comparison of the mean (n = 5 replicates) viral titers obtained in the neutralization effectiveness studies (Supplemental Fig. S1) was performed using a non-parametric unpaired *t*-test, with statistical significance set at *P* < 0.05.

## Supplementary information

Supplemental information
