# Supplementary material for: Effectiveness of Dettol Antiseptic Liquid for Inactivation of Ebola Virus in Suspension
Source: Sci Rep. 2019 Apr 29;9:6590. doi: 10.1038/s41598-019-42386-5 (PMC6488606; doi:10.1038/s41598-019-42386-5)
Supplement: Supplementary file 1 — Supplemental information [file 41598_2019_42386_MOESM1_ESM.docx]

**Supplemental Material for**

**Effectiveness of Dettol Antiseptic Liquid for Inactivation of Ebola Virus in Suspension**

**Todd A. Cutts, M. Khalid Ijaz, Raymond W. Nims, Joseph R. Rubino, Steven S. Theriault**

**Supplemental Figure S1.** Ability of Letheen broth to neutralize the EBOV/Mak-inactivating effects of DAL dilutions of 1:10, 1:20, and 1:40 in hard water. TCID_50_/mL, tissue culture infectious dose_50_/mL. No significant differences were observed by unpaired *t*-test (*P* < 0.05) between Virus alone, Virus + Neutralizer, and Virus + Neutralizer + Disinfectant conditions for each DAL dilution.


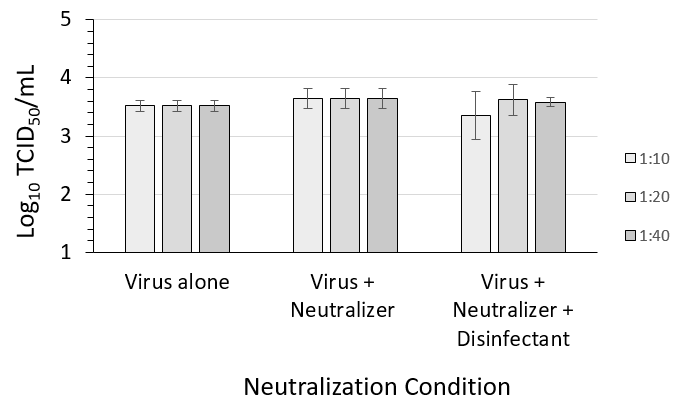


**Supplemental Table S1.** Cytotoxicity to Vero E6 cells resulting from exposure to DAL dilutions of 1:10, 1:20, and 1:40 in hard water. The lowest dilution of the post-neutralization solution which did not cause cytotoxicity to the Vero-E6 cells is shown (10^0^ = undiluted; 10^-1^ = 1:10 dilution)

| Test Condition | 1:10 DAL | | | 1:20 DAL | | | 1:40 DAL | | |
| --- | --- | --- | --- | --- | --- | --- | --- | --- | --- |
|  | Lot 1 | Lot 2 | Lot 3 | Lot 1 | Lot 2 | Lot 3 | Lot 1 | Lot 2 | Lot 3 |
| Negative control | 10^0^ | 10^0^ | 10^0^ | 10^0^ | 10^0^ | 10^0^ | 10^0^ | 10^0^ | 10^0^ |
| Neutralizer + DAL | 10^-1^ | 10^-1^ | 10^-1^ | 10^-1^ | 10^-1^ | 10^-1^ | 10^-1^ | 10^-1^ | 10^-1^ |
